# Molecular identification of hookworm infection in humans, dogs and cats in Lao People’s Democratic Republic

**DOI:** 10.1186/s40249-025-01403-8

**Published:** 2026-01-20

**Authors:** Somphou Sayasone, Chomseng Norkhankham, Sysouphanh Many, Anousin Homsana, Tawin Inpankaew, Peter Odermatt

**Affiliations:** 1 Lao Tropical and Public Health Institute, Ministry of Health, Vientiane Capital, Lao People’s Democratic Republic; 2 Department of Parasitology, Faculty of Veterinary Medicine, Kasetsart University, Bangkok, Thailand; 3 Epidemiology and Public Health Department, Swiss Tropical and Public Health Institute, Allschwil, Switzerland; 4 University of Basel, Basel, Switzerland

**Keywords:** Hookworm, Species identification, Zoonotic hookworm, Dog, Lao PDR

## Abstract

**Background:**

Hookworm infection remains a major public health concern in the Lao People’s Democratic Republic (Lao PDR), where it is endemic nationwide and particularly prevalent in remote communities. However, information regarding species-specific identification is limited. This study aimed to determine the prevalence of hookworm infection and identify the infecting species among humans, dogs, and cats in both northern and southern regions of the Lao PDR.

**Methods:**

A cross-sectional study was conducted between May and July 2023 in Luangprabang Province (northern Lao PDR) and Champasak Province (southern Lao PDR). All residents aged 18 years and older who were present during the survey period and met the inclusion criteria were enrolled. In addition, dogs and cats owned by enrolled participants and/or their household members were included. Hookworm infection was detected by identifying eggs in preserved stool samples using the formalin-ethyl acetate concentration technique (FECT). Species identification was performed using polymerase chain reaction (PCR) targeting the internal transcribed spacer (*ITS*) region to amplify *Necator americanus* (485 bp) and *Ancylostoma* spp. (380 bp), followed by sequencing analysis of stool samples preserved in 70% ethanol. Descriptive statistics summarized hookworm prevalence in humans, dogs, and cats. Chi-square, Wilcoxon rank-sum, and Kruskal–Wallis tests compared rates and means, with significance defined as *P* < 0.05.

**Results:**

A total of 382 human participants, along with 37 dogs and 9 cats, completed all study procedures and were included in the analysis. Examination of preserved stool samples using FECT detected hookworm infection in 24.1% of humans (southern region: 36.6%; northern region: 11.8%) and in 76.1% of dogs and cats combined, with the highest prevalence observed in the southern region (92.6%). Molecular analysis identified *N. americanus* as the predominant species in humans (64.6%), followed by *A. ceylanicum* (20.5%). Among dogs and cats, *N. americanus* and *A. caninum* were each detected in 39.1% of samples, while *A. ceylanicum* accounted for 13.0%.

**Conclusions:**

This study demonstrates a high prevalence of zoonotic hookworm infections in humans, dogs, and cats, highlighting the potential for interspecies transmission that complicates current prevention and control measures. Implementation of integrated control strategies—such as the One Health approach—that simultaneously address human and animal reservoirs is essential for achieving effective and sustainable hookworm control in the Lao PDR.

**Supplementary Information:**

The online version contains supplementary material available at 10.1186/s40249-025-01403-8.

## Background

Hookworm, a nematode intestinal parasite, is a common soil-transmitted helminth infection that poses a significant health problem worldwide, particularly in rural areas of tropical and subtropical developing countries [1]. Infection with hookworm primarily occurs through skin penetration upon contact with the infective stage of the larvae in contaminated soil [2, 3]. It is one of the most prevalent chronic infections, affecting people in remote settings with poor hygiene, inadequate sanitation, and limited access to safe water [4]. Although hookworm infection rarely results in death, it can lead to intestinal blood loss, poor iron status, and potentially cause iron deficiency anemia [1]. Importantly, the infection may impair growth and cognitive development, particularly in children and women of reproductive age [1, 5].

*Necator americanus* and *Ancylostoma duodenale* are the predominant human hookworm species globally [2, 3]. Zoonotic hookworms, including *Ancylostoma ceylanicum* and *Ancylostoma caninum*, predominantly infect dogs and cats but have occasionally been reported in humans [6, 7]. The presence of these zoonotic hookworms complicates diagnosis. While parasitological methods widely employed in surveillance and control programs in developing countries can detect hookworm eggs in stool samples, species identification requires advanced molecular techniques, such as polymerase chain reaction (PCR) and sequencing [7, 8]. Consequently, species identification of hookworms is rarely performed in most resource-constrained areas.

In Lao People’s Democratic Republic (Lao PDR), hookworm infection remains a significant public health problem. The nationwide prevalence in adults is approximately 21.6%, with particularly high rates in remote areas where most inhabitants engage in subsistence agricultural farming with high contact with soil during farming activities and limited access to improved sanitation and safe water [9]. Although this study showed highly prevalent of hookworm infection, but its species distribution and cross-species infection were unidentified. Decades ago, few studies suggested that *N. americanus* is a predominant hookworm infection and *A.* *ceylanicum* is the zoonotic species identified among study participants [6]. However, there is a lack of recent assessments on species distribution. This study aims to identify hookworm species circulating in Luangprabang and Champasak provinces of the Lao PDR, where our previous national survey had documented a high prevalence of hookworm infection [9]. The results generate from this study would provide evidence to improve the national control programme.

## Methods

### Study area and population

We conducted a cross-sectional study between May and July 2023 to identify hookworm species circulating in Luangprabang and Champasak provinces of the Lao PDR. These provinces were selected based on findings from our previous national survey, which had documented a high prevalence of hookworm infection [9]. Luangprabang is the largest province in northern Lao PDR. It comprises 12 districts and has a population of 431,889 [10]. Our study was conducted in the Nambak district, approximately 100 km north of Luangprabang city (geocoordinates: 20.62° N, 102.47° E). Champasak province is the largest province in the southern part of Lao PDR. It encompasses 10 districts and has a total population of 694,023 [10]. Our fieldwork was conducted in the Batiengchaleunsouk district, which is located approximately 10 km from Pakse city (geocoordinates: 15.25° N, 105.95° E).

### Sampling procedure

From each district, two villages known to have a high prevalence of hookworm (two villages in Nambak District, Luangprabang Province, and two villages in Batiengchaleunsouk District, Champasak Province), were selected as study villages [9]. We enrolled all adults aged 18 and older who were present in the selected villages on the survey day to our study. Only participants who agreed to sign an informed consent form were enrolled. Enrolled participants provided two stool samples for parasitological examination. Additionally, all dogs and cats owned by the study participants or their household members were included in the study for parasitological assessment.

### Sample size calculation

The sample size calculation for this study followed the formula: $\mathrm{N}=\frac{P\left(P-1\right){Z}^{2}}{{E}^{2}}$, with the components: (i) *P* (prevalence), the prevalence was 21%, as determined from our previous study [9], (ii) *Z* (level of confidence) set at 95% and E (margin of error) was 0.05. Based on these parameters, the initial sample size required was 255 individuals. To account for potential dropouts or non-compliance during the study, we added 20%. Consequently, a total sample size of 306 participants was targeted.

### Field and laboratory procedures

The field team selected a temporary research station as a working place in each village; usually a temple, village office, or school. A meeting was organized with village authorities and villagers to explain the objective and plan the field activities. The villagers who met the study criteria and were willing to participate in the study were invited to register. Later, the research team assigned a unique identity number to each registered villager and arranged an appointment with her/him on the following day for the written informed consent and enrolment process. Each study participant received a pre-labelled container (30 ml) with a unique ID. After signing an informed consent form, a laboratory technician provided clear instructions on how to collect the stool sample, where to return it, and when. For each stool sample, we aliquoted 2 g into a 15 ml tube containing 10 ml of sodium acetate, acetic acid, and formalin (SAF) solution for parasitological analysis. Additionally, 1 g of the sample was fixed in 70% ethanol for polymerase chain reaction (PCR) analysis.

In parallel with human sample collection, each dog and cat owned by a study participant or a member of their household was confined in an individual cage, clearly labelled with a unique identification number. The dog and/or cat was given a gel Unison enema (Sodium chloride 15%) and left it in the cage until it defecated. A sample of 2 g was collected and fixed in SAF for parasitological analysis, and 1 g was fixed in 70% ethanol for PCR analysis.

The samples fixed in SAF solution were transported to the laboratory at the Lao Tropical and Public Health Institute (Lao TPHI) for parasitological analysis using the formalin-ethyl-acetate concentration technique (FECT) [11, 12]. All eggs of parasites detected under the light microscope were identified and recorded separately.

Samples fixed in 70% ethanol were sent to the Department of Parasitology at the Faculty of Veterinary Medicine, Kasetsart University, Bangkok, Thailand, for identification of the hookworm species using PCR.

### DNA extraction

The Genomic DNA from the human and animal stool samples was extracted directly from 200 mg stool samples and Glass Beads X using a commercial extraction kit, E.Z.N.A® Stool DNA kit (Omega Bio-tek, Inc., Norcross, Georgia, USA), following the manufacturer's instructions. The final eluted volume of 100 µl was stored at − 20 °C until it was analysed by conventional polymerase chain reaction.

### Molecular characterization

#### In humans

All 92 samples confirmed as hookworm-positive by microscopic analysis using FECT were subjected to molecular analysis. A subset of 35 microscopically negative samples (10% per province) was included alongside the confirmed hookworm-positive samples to assess potential false-negative results by microscopy, particularly in cases of low-intensity infections. Genomic DNA was extracted from all samples and screened for *Necator americanus* and *Ancylostoma* spp. infections using polymerase chain reaction. This method amplified a fragment of 380–485 bp from the internal transcribed spacer (ITS)-1, 5.8S, and ITS-2 regions, as described by Traub et al., 2008 (Table 1) [8]. The suspected *N. americanus* (485 bp) (Figure S1) and *Ancylostoma* spp. (380 bp) (Figure S2) band were cut and purified using the E.Z.N.A® Gel Extraction kit (Omega Bio-tek, Inc., Norcross, Georgia, USA), following the manufacturer’s instructions. The purified samples were then sent to Macrogen® in Gangnam-gu, Seoul, Republic of Korea, for sequencing. The sequences were analysed for similarity using the Basic Local Alignment Search Tool (BLAST) (https://blast.ncbi.nlm.nih.gov/Blast.cgi).

**Table 1:** Primer used for PCRs for the characterization of hookworms in humans and domestic animals (dogs and cats)

Tested species	Primers	Parasite	Product (bp)	References
Humans	RTHW1F RTHW1R	*Necator americanus* *Ancylostoma* spp.	485 380	Traub et al. [13]
Dogs/Cats	RTGHF1 RTABCR1	*Ancylostoma caninum* *Ancylostoma ceylanicum*	545	Traub et al. [8], Palmer et al. [14]; Inpankaew et al. [7]
Dogs/Cats	RTGHF1 RTAYR1	*Ancylostoma braziliense*	673	Traub et al. [8], Palmer et al. [14]

*PCR* polymerase chain reaction

#### In dogs and cats

All 46 stool samples collected from dogs and cats were molecularly analysed using diagnostic PCR-restriction fragment length polymorphism (RFLP) to characterize hookworm species, following previously described protocols [7]. Primers sets listed in Table 1 were used to amplify a region of the internal transcribed spacer (ITS)-1, 5.8S, and ITS-2. The amplified PCR products of RTGHF1–RTABCR1 were digested with *Rsa*I to differentiate *Ancylostoma tubaeforme* from *A. ceylanicum* and *A. caninum. HinF*1 was used to distinguish *A. caninum* from *A. ceylanicum*. RFLP profiles obtained from each sample were compared with established reference patterns for each hookworm species [13, 14].

Representative sequences from both humans and animal samples were deposited in GenBank under accession numbers PX497935–PX497937 (*A. ceylanicum*), and PX497932-PX497934 (*A. caninum*).

### Data management and analysis

Data was digitally collected using tablets. The questionnaire was developed in the CommCare server (www.commcarehq.org) to capture demographic information from study participants, including age, sex, gender, ethnicity, educational level, occupation, availability of a household latrine and ownership of pets (dogs or cats). The Commcare ODK application was installed on the tablet for daily field data collection. Collected data were synchronized to the CommCare server and downloaded into an Excel spreadsheet for consistency and completeness checks. The double-checked data was transferred to STATA, version 14 (Stata Corporation, College Station, TX, USA) for analysis. Frequencies described hookworm prevalence in humans, dogs, and cats. The mean summarized the intensity of hookworm infection, presented as eggs per gram of stool (EPG). Associations between infection and demographic data were analysed using Chi-squared tests. Wilcoxon rank-sum and Kruskal–Wallis tests were used to compare means where appropriate. *P*-value of less than 0.05 was considered statistically significant.

## Results

### Study subjects

**Fig. 1:**
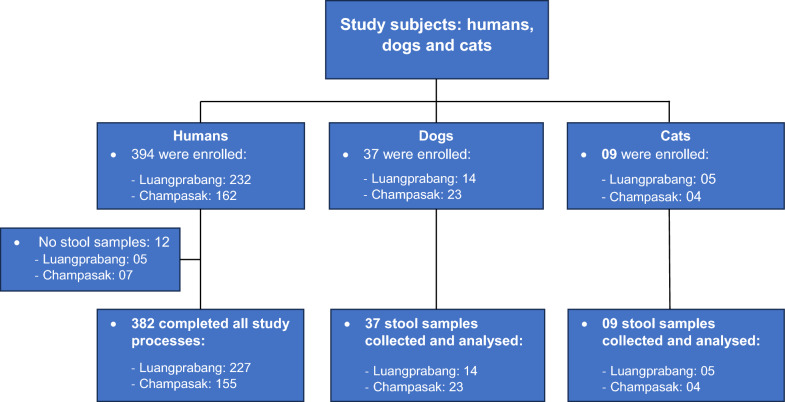
Study subjects (humans, dogs and cats) enrolled in the study by province

In total, 394 villagers, 37 dogs and 9 cats were initial enrolled in the study. Of these, 12 villagers failed to submit stool samples for parasitological analysis (5 from Luangprabang and 7 from Champasak). Consequently, 382 villagers (Luangprabang: 227; Champasak: 162), 37 dogs (Luangprabang: 14; Champasak: 23), and 9 cats (Luangprabang: 5; Champasak: 4) successfully completed all study procedures and were included in the final data analysis (Fig. 1). Among the 382 villagers, gender distribution was comparable between Luangprabang and Champasak (*P* = 0.597). The median age was slightly higher in Luangprabang (55 years) than in Champasak (51 years). The age group analysis showed a statistically significant difference (*P* = 0.049), with younger participant profile in Champasak. Ethnic composition differed significantly between the two provinces (*P* < 0.001): Lao-Tai participants predominated in Luangprabang (46.3%), whereas ethnic minorities constituted the majority in Champasak (88.4%). Access to sanitation facilities also differed significantly (*P* = 0.006), with nearly universal latrine coverage in Luangprabang (99.6%) compared to lower access in Champasak (95.5%) (Table 2).

**Table 2:** Sociodemographic characteristics of 382 study participants, stratified by province

Variables	Overall *n* = 382, % (*n*)	Luangprabang *n* = 227, % (*n*)	Champasak *n* = 155, % (*n*)	${x}^{2}$	*P*-value
Gender					
Male	40.3 (154)	41.4 (94)	38.7 (60)		
Female	59.7 (228)	58.6 (133)	61.3 (95)	0.28	0.597
Age in year					
Median age (IQR)	54 (40–60)	55 (43–60)	51 (36–60)		
Age group (years)					
18–35	17.3 (66)	13.2 (30)	23.2 (36)		
36–49	24.1 (92)	23.8 (54)	24.5 (38)		
50–59	20.9 (80)	23.8 (54)	17.8 (26)		
≥ 60	37.7 (144)	39.2 (89)	3.5 (55)	7.86	0.049
Ethnicity					
Lao-Tai	31.4 (120)	46.3 (101)	11.6 (19)		
Minorities	68.6 (262)	53.7 (117)	88.4 (145)	44.42	< 0.001
Education level					
Illiterate	16.5 (63)	14.2 (31)	19.5 (32)		
Primary school	45.3 (173)	64.8 (102)	43.3 (71)		
Secondary/high school	33.5 (128)	14.4 (75)	32.3 (53)		
College or higher	4.7 (18)	4.6 (10)	4.9 (8)	1.13	0.771
Occupation					
Employees	17.8 (68)	16.1 (25)	18.9 (43)		
Farmers	82.2 (314)	83.9 (130)	81.1 (184)	0.49	0.480
Having pets at home					
No	63.9 (244)	49.7 (77)	73.6 (167)		
Yes	36.1 (138)	50.3 (78)	26.4 (60)	22.78	< 0.001
Latrine at home					
No	2.1 (8)	0.4 (1)	4.5 (7)		
Yes	97.9 (374)	99.6 (226)	95.5 (148)	7.46	0.006

^*^ *P-*value was obtained from Chi-square test; *P-*value < 0.05 was counted as statistical significance

### Intestinal parasitic detection in FECT

Among the 382 villagers, analysis of preserved stool samples using the formalin-ether concentration technique (FECT) revealed an overall prevalence of intestinal helminth infections of 36.6%. Hookworm was the most prevalent parasite, detected in 24.1% of samples, followed by *Strongyloides stercoralis* (10.7%) and *Opisthorchis viverrini* (6.0%). The prevalence of these intestinal parasites was significantly higher in Campasak compared to Laungprabang: hookworm (36.6% vs 16.5%, *P* < 0.001), *S. stercoralis* (16.5% vs 6.4%, *P* = 0.002) and *O. viverrini* (9.7% vs 3.2%, *P* = 0.008), respectively. Other intestinal parasites, including *Ascaris lumbricoides*, *Trichuris trichiura*, *Taenia* spp., and *Giardia intestinalis*, were identified at prevalences below 5%, with no statistically significant differences (*P* > 0.05) between province (Table 3).

**Table 3:** Prevalence of intestinal parasitic infections among 382 study participants detected in the stool analysis using formalin-ethyl acetate concentration technique stratified by province

Parasite	Overall *n* (%), *n* = 382	Luangprabang *n* (%), *n* = 227	Champasak *n* (%), *n* = 155	${x}^{2}$	*P*-value*
Nematodes					
Hookworm	24.1 (96)	16.5 (36)	36.6 (60)	20.04	< 0.001
*Strongyloides stercoralis*	10.7 (41)	6.4 (14)	16.5 (27)	9.85	0.002
*Trichuris trichiura*	1.6 (6)	2.4 (4)	0.9 (2)	1.40	0.236
*Ascarid lumbricoides*	1.0 (4)	0.4 (1)	1.9 (3)	1.69	0.193
*Enterobius vermicularis*	0.5 (2)	0	2 (1.2)	2.67	0.102
Trematodes					
*Opisthorchis viverrini*	6.0 (23)	3.2 (7)	9.7 (16)	7.09	0.008
Big trematode egg	0.3 (1)	0.5 (1)	0	0.75	0.385
Cestodes					
*Taenia* spp.	2.9 (11)	1.8 (4)	4.3 (7)	1.95	0.162
Protozoal					
*Giardia intestinalis*	0.5 (2)	0.4 (1)	0.6 (1)	0.23	0.895

^*^ Statistical significance if *P-*value < 0.05 based on Chi-square test

Regarding hookworm infection, women harboured a slightly higher prevalence (25.0%) and mean intensity of infection (6.6 EPG vs. 2.1 EPG) compared to men (22.7%; 2.1 EPG), although these differences were not statistically significant (*P* > 0.05). The highest prevalence was observed among individuals aged 18–35 years (27.3%). Notably, ethnic minority groups demonstrated significantly higher prevalence (27.1% vs. 17.5%, *P* = 0.042) and intensity of infection (6.4 EPG vs. 1.4 EPG, *P* = 0.025) compared to the Lao-Tai group (Table 4).

**Table 4:** Prevalence and intensity of hookworm infections among 382 study participants detected in stool analysis using formalin-ethyl acetate concentration technique

Indicators	No. of sample analysis	Prevalence		Intensity	
		% (*n*)	*P*-value	Mean EPG (95% *CI*)	*P*-value
Gender					
Male	154	22.7 (35)		2.1 (1.1–3.2)	
Female	228	25.0 (57)	0.610^a^	6.6 (2.8–10.4)	0.449^a^
Age group					
18–35	66	27.3 (18)		4.8 (1.5–8.2)	
36–49	92	22.8 (21)		5.0 (0.7–9.3)	
50–59	80	21.3 (17)		1.9 (0.2–3.6)	
≥ 60	144	25.0 (36)	0.834^a^	6.3 (1.1–11.4)	0.657^b^
Ethnicity					
Lao-Tai	120	17.5 (21)		1.4 (0.5–2.3)	
Minorities	262	27.1 (71)	0.042^a^	6.4 (3.0–9.7)	0.025^c^

*EPG* Mean egg per gram of stool, *CI* Confidence interval

a) *P-*value obtained from Chi-square test

b) *P-*value obtained from Wilcoxon rank-sum test

c) *P-*value obtained from Kruskal-Wallis test

For dogs and cats, the analysis of preserved stool samples revealed intestinal parasitic infections in 87.0% (40 out of 46 animals) of the animals. Hookworm was the most prevalent parasite (76.1%), with significantly higher prevalence (*P* = 0.002) in Champasak (92.6%) compared to Luangprabang (52.6%). Other intestinal parasites, included *Trichuris* spp. (10.9%), *Ascaris* spp. (10.9%), *Strongyloides stercoralis* (8.7%), *Enterobius vermicularis* (2.2%), *Taenia* spp.*,* (4.3%), *Dipylidium caninum* (4.3%), large trematode eggs (2.2%) and *Toxoplasma gondii* (6.5%), showed no statistically significant differences between provinces (*P* > 0.05) (Table 5).

**Table 5:** Prevalence of intestinal helminth infections among dogs and cats detected in ormalin-ethyl acetate concentration technique, *n* = 46

Intestinal parasites	Overall *n* = 46, % (*n*)	Luangprabang *n*, % (*n*)	Champasak % (*n*)	${x}^{2}$	*P*-value
Nematodes					
Hookworm	76.1 (35)	52.6 (10)	92.6 (25)	9.79	0.002
*Trichuris spp*.	10.9 (5)	10.5 (2)	11.1 (3)	< 0.01	0.950
*Ascaris lumbricoides*	10.9 (5)	15.8 (3)	7.4 (2)	0.81	0.368
*Strongyloides spp*.	8.7 (4)	15.8 (3)	3.7 (1)	2.05	0.152
*Enterobius vermicularis*	2.2 (1)	0	3.7 (1)	0.72	0.396
Cestodes					
*Taenia spp.*	4.3 (2)	10.5 (2)	0	2.97	0.085
*Dipylidium caninum*	4.3 (2)	0	7.4 (2)	1.47	0.225
Trematodes					
Large trematode eggs	2.2 (1)	0	3.7 (1)	0.71	0.396
Protozoa					
*Toxoplasma gondii*	6.5 (3)	0	11.1 (3)	2.26	0.133

^*^ *P-*value was obtained from Chi-square test

### Hookworm species identification

A sub-sample of 127 human stool samples, comprising 92 microscopically confirmed hookworm-positive cases and 35 negatives, was subjected to PCR amplification for species identification. PCR analysis successfully amplified hookworm DNA in 98 samples (77.2%), including 88 from microscopy-confirmed infections and 10 from microscopy-negative samples. The remaining 29 samples (22.8%) failed to yield species-specific sequences, consisting of 4 microscopy-positive and 25 microscopy-negative cases. Table 6 summarizes the molecular characterization of hookworm species among human samples, stratified by province. Sequencing of the 98 PCR-positive samples identified two distinct hookworm species. *Necator americanus*, a human-specific species, was predominant, detected in 82 of 98 samples (83.7%). Its prevalence was significantly higher in Champasak compared to Luangprabang (90.9% vs 68.8%, *P* = 0.005). *A. ceylanicum* was identified in 26 of 98 samples (26.5%), with a higher prevalence in Luangprabang than in Champasak (53.1% vs. 13.6%, *P* < 0.001). Single-species infections, either *N. americanus* or *A. ceylanicum,* accounted for 89.8% (88/98), while double infections were observed in 10.2% (10/98), respectively. No co-infections of triple or more species were identified in these samples.

**Table 6:** Molecular characterization of hookworm species among 98 PCR-positive samples, stratified by province

Hookworm species	Overall, % (*n*)	Luangprabang, % (*n*)	Champasak, % (*n*)	${x}^{2}$	*P*-value
	*n* = 98	*n* = 32	*n* = 66		
*Necator americanus*					
No	16.3 (16)	31.2 (10)	9.1 (6)		
Yes	83.7 (82)	68.8 (22)	90.9 (60)	7.75	0.005
*Ancylostoma ceylanicum*					
No	73.5 (72)	46.9 (15)	86.4 (57)		
Yes	26.5 (26)	53.1 (17)	13.6 (9)	17.24	< 0.001
Co-infection					
*Necator americanus*	73.5 (72)	46.9 (15)	86.4 (57)		
*Ancylsotoma ceylanicum*	16.3 (16)	31.2 (10)	9.1 (6)		
Double infections	10.2 (10)	21.9 (7)	4.5 (3)	17.39	< 0.001

*PCR* Polymerase Chain Reaction

^*^ P-value obtained from Chi-square test; *P-*value < 0.05 was counted as significant

**Fig. 2:**
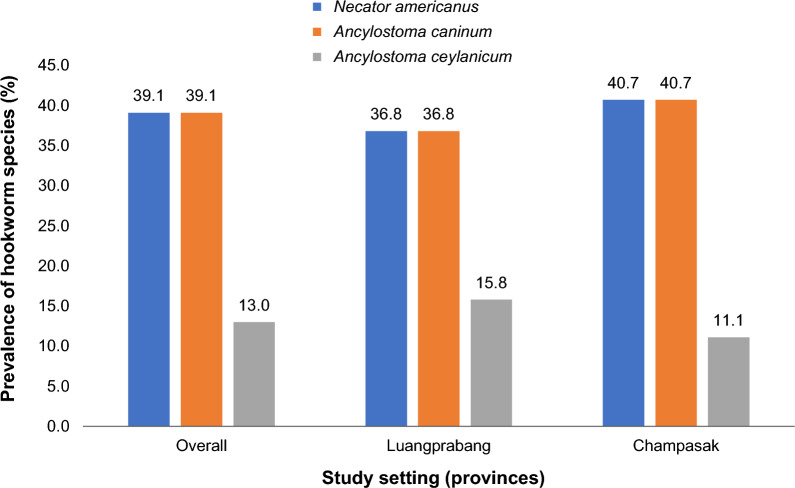
Hookworm species characterization among 46 dogs and cats by provinces

Among the 46 animal stool samples subjected to PCR analysis, 35 were microscopically confirmed as hookworm-positive, while 11 samples tested negative. PCR amplification successfully detected hookworm DNA in 34 samples (77.9%), including 30 of the 35 microscopy-confirmed positives and 4 of the 11 microscopy-negative samples. In five microscopy-positive samples (14.3%), the PCR amplification failed to identify products suitable for species identification. Sequencing of the 34 PCR-positive samples revealed three hookworm species. *N. americanus* and *A. caninum* were each detected in 18 samples (39.1%), with the highest prevalence observed in Champasak Province (40.7% each), while *A. ceylanicum* was detected in 6 of the 46 samples (13.0%), showing its highest prevalence in Luangprabang Province (15.8%) (Fig. 2). *A. ceylanicum* was exclusively identified in 6 of the 39 canine hosts (15.4%). In contrast, *N. americanus* and *A. caninum* were detected in both dogs and cats, with prevalence rates of 41.0% and 28.6%, respectively. Single species infections accounted for 56.5% of the sequenced samples. All feline samples were positive for only a single hookworm species, either *N. americanus* (28.6%) or *A. caninum* (28.6%)*.* Double-species infections were observed exclusively in canine samples, involving *N. americanus* with either *A. caninum* (15.2%) or *A. ceylanicum* (2.2%). No triple co-infections were identified in these animal stool samples (Table 7).

**Table 7:** Hookworm species characterization among dogs and cats enrolled in the study

Species characterization	Overall *n* (%), *n* = 46	Dogs *n* (%), *n* = 39	Cats *n* (%), *n* = 7
*Necator americanus*	39.1 (18)	41.0 (16)	28.6 (2)
*Ancylostoma caninum*	39.1 (18)	41.0 (16)	28.6 (2)
*Ancylostoma ceylanicum*	13.0 (6)	15.4 (6)	0
Single infection			
No infection	26.1 (12)	23.1 (9)	42.8 (3)
Only *Necator americanus*	15.2 (7)	12.8 (5)	28.6 (2)
Only *Ancylostoma caninum*	28.3 (13)	28.2 (11)	28.6 (2)
* Only Ancylostoma ceylanicum*	13.0 (6)	15.4 (6)	0
Double infections			
*Necator americanus* + *Ancylostoma caninum*	15.2 (7)	17.9 (7)	0
*Necator americanus* + *Ancylostoma ceylanicum*	2.2 (1)	2.6 (1)	0

Single infection: sequencing analysis identified only *A. americanus* or *A. Ceylanicum* or *A. caninum* in the samples

Double infections: sequencing analysis identified *N. americanus* and *A. Ceylanicum or N. americanus* and *A. caninum* in the samples

Overall infections: sequencing analysis identified any *N. americanus* or *A. Ceylanicum* or *A. caninum* in the samples

## Discussion

Hookworm infection remains a significant public health concern in the Lao PDR, with high endemicity reported nationwide [9]. Despite its considerable burden, species-level data, and information on potential animal reservoirs have been limited, hindering the development of targeted and effective interventions. To address these gaps, the current study employed both parasitological and molecular methods to characterize hookworm infections in humans, dogs, and cats in two endemic provinces.

Analysis of stool samples preserved in SAF and examined using the FECT revealed the high prevalence of hookworm at 24.1% in humans and 76.1% in dogs and cats. These findings align with a previous studies conducted a decade earlier in northern provinces, which also reported the high prevalence rates of 46.3% in humans and 89.5% in dogs based on conventional parasitological techniques [6]. Additional studies in Champasak province reported hookworm prevalence of 62.2% in dogs [6] and 48.8% in humans [15]. However, in these two subsequent studies, species-level identification was not performed. The consistently high prevalence across host populations underscores the enduring public health and veterinary importance of hookworm in Lao PDR and suggests a possible zoonotic interface between human and animal reservoirs. This study observed the geographical variation in prevalence. Champasak province exhibited significantly higher rates of hookworm infection compared to Luangprabang, both in humans (36.6% vs 16.5%, *P* < 0.001) and in animals (92.6% vs 52.6%, *P* = 0.002). Several ecological and environmental factors may contribute to this disparity. Champasak, located in the lowland Mekong basin, is characterized by a warmer, more humid climate and extensive agricultural activity, conditions that favour the survival and development of hookworm larvae in soil. In contrast, Luangprabang’s cooler, more mountainous environment may limit larval development and reduce transmission intensity. Although this study did not directly assess risk factors associated with infection, previous studies have identified key determinants, including household socio-economic status, participation in agricultural work, and sanitation practices, as significantly associated with infection [9, 15].

In this study, all human and animal stool samples preserved in 70% ethanol and testing positive by microscopy were subjected to PCR and sequencing analyses to characterize hookworm species. Additionally, a subset of microscopy-negative samples was also analysed to investigate potential false negatives, which can occur when using parasitological methods, particularly in cases of low infection intensity [16]. Sequencing analysis identified three hookworm species in preserved human and animal samples: *Necator americanus,* a human specific species and two zoonotic species, *Ancylostoma ceylanicum* and *A. caninum*. *N. americanus* was predominant species detected across all three hosts, including humans, dogs and cats. *A. ceylanicum* was identified in both human and dog samples, while *A. caninum* was exclusively identified in animal samples (dogs and cats). The presence of *N. americanus* and *A. ceylanicum* in both human and animal hosts underscore their public health and veterinary significance in Lao PDR and supports growing evidence of cross-species transmission at the human–animal interface. *A. ceylanium* was the principal zoonotic hookworm species infected humans in Lao PDR. This finding aligns with a recent review and other studies conducted in Southeast Asia indicating that *A. ceylanicum* is the predominant zoonotic species responsible for human infections in the region [6, 7, 17, 18].

Interestingly, the molecular analysis of microscopy-negative samples identified hookworm DNA in 10 of the 35 human samples and 4 of the 11 animal samples, indicating false-negative results by conventional parasitological methods. These findings highlight the superior sensitivity of molecular diagnostics in detecting infections and demonstrate their value in providing a more accurate assessment of infection burden compared with traditional parasitological techniques. This observation is consistent with previous studies reporting higher sensitivity of molecular methods for detecting soil-transmitted helminth infections, including hookworm than conventional parasitological techniques [11, 19–21]. However, it is noteworthy that 4 of the 92 human samples and 5 of the 35 animal samples confirmed as hookworm-positive by FECT were molecularly negative. Such discrepancies between parasitological and molecular results have been reported previously and may be attributed to several factors, including DNA degradation in ethanol-preserved samples, the presence of PCR inhibitors in stool, or low worm burden below the detection threshold of the assay [21, 22]. In some cases, microscopy may detect eggs that are no longer viable or contain insufficient DNA for amplification. Conversely, misidentification of hookworm eggs under microscopy cannot be entirely excluded, particularly in regions such as Lao PDR where human *Trichostrongylus colubriformis* infections have been documented in rural communities [23, 24]. The eggs of *Trichostrongylus* spp. are morphologically similar to those of hookworms, and this resemblance can lead to diagnostic confusion when relying solely on parasitological methods, which may consequently explain some of the PCR-negative results.

Furthermore, the sequencing analysis revealed double-species hookworm infections in approximately one-tenth of human samples and one-fifth of animal samples. These co-infections have important implications for both epidemiological analysis and diagnostic interpretation [6, 20]. First, co-infections complicate prevalence estimates when species cannot be distinguished by microscopy, leading to potential underestimation of zoonotic species such as *A. ceylanicum,* which is highly prevalence in both humans and animals in the region. Second, the presence of more than one species within a single specimen may influence amplification efficiency in molecular analyses, occasionally resulting in preferential detection of the dominant species and underrepresentation of the secondary species. Such challenges highlight the necessity of employing molecular methods capable of resolving mixed-species infections to ensure accurate species attribution, which is critical for understanding transmission dynamics and designing effective integrated control strategies.

This study was conducted in purposively selected villages with known high hookworm prevalence [9]. Therefore, the reported prevalence in humans, dogs, and cats should not be generalized to the wider population. The relatively small number of animal samples also limits the reliability of host-specific analyses. In addition, some discrepancies between microscopy and molecular results may reflect sample preservation issues, PCR inhibition, or misidentification of morphologically similar eggs such as *Trichostrongylus* spp. Future studies with larger, representative animals are needed to clarify host roles in the transmission dynamic, which may be to inform evidence-based policy and the design of sustainable integrated interventions.

## Conclusions

This study demonstrates the added value of molecular diagnostics in elucidating the epidemiology of hookworm infections in Lao PDR. PCR not only detected infections missed by microscopy but also enabled species-level identification, revealing the co-circulation of *Necator americanus*, *Ancylostoma ceylanicum*, and *A. caninum* in both humans and animals. The detection of double-species infections in approximately one-tenth of human samples and one-fifth of animal samples highlights the complexity of transmission dynamics and the limitations of relying solely on conventional parasitological methods. Importantly, the identification of zoonotic *A. ceylanicum* in both humans and dogs underscores the role of animal reservoirs and the necessity of a One Health approach to surveillance and control. Together, these findings emphasize that accurate species attribution is critical for refining prevalence estimates, understanding transmission pathways, and designing effective, integrated intervention strategies.

## Supplementary Information

Supplementary Material 1.

Supplementary Material 2.

## Data Availability

Data is available at the Lao TPHI and collaborators’ institutions and is fully accessible to all co-authors. Data can be shared with other institutions and researchers upon reasonable request.

## Abbreviations

Lao PDR: Lao People’s Democratic Republic
EPG: Eggs per gram
FECT: Formalin-ethyl acetate concentration technique
PCR: Polymerase chain reaction
NECHR: National Ethics Committee for Health Research
*CI*: Confidence interval
