# Supplementary material for: Molecular identification of hookworm infection in humans, dogs and cats in Lao People’s Democratic Republic
Source: Infect Dis Poverty. 2026 Jan 20;15:13. doi: 10.1186/s40249-025-01403-8 (PMC12817831; doi:10.1186/s40249-025-01403-8)
Supplement: Supplementary file 2 — Supplementary Material 2. [file 40249_2025_1403_MOESM2_ESM.docx]

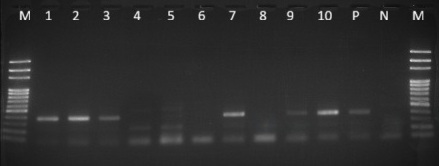


**Figure S2**. Gel electrophoresis of ITS-2 PCR products for the detection of *Ancylostoma* *spp.* PCR targeting the ITS-2 region generated a specific band of approximately 380 bp for *Ancylostoma* *spp.* Lane M: 100 bp DNA ladder; Lanes 1–10: field samples showing positive amplification at ~380 bp; Lane P: positive control DNA of *Ancylostoma* *spp.*; Lane N: negative control; Lane M: 100 bp DNA ladder.
